# Isokotomolide A from Cinnamomum kotoense Induce Melanoma Autophagy and Apoptosis *In Vivo* and *In Vitro*

**DOI:** 10.1155/2020/3425147

**Published:** 2020-09-26

**Authors:** Jian Li, Chung-Yi Chen, Jyun Yin Huang, Lin Wang, Zixuan Xu, Wenyi Kang, Miao-Hsia Lin, Hui-Min David Wang

**Affiliations:** ^1^College of Food and Biological Engineering, Jimei University, Xiamen 361021, China; ^2^Department of Nutrition and Health Science, School of Medical and Health Sciences, Fooyin University, Kaohsiung 831, Taiwan; ^3^Graduate Institute of Biomedical Engineering, National Chung Hsing University, Taichung 402, Taiwan; ^4^College of Chemistry & Pharmacy, Northwest A&F University, Yangling, Shaanxi 712100, China; ^5^Joint International Research Laboratory of Food & Medicine Resource Function, Henan Province, Henan University, Kaifeng 475004, China; ^6^Graduate Institute of Microbiology, College of Medicine National Taiwan University, Taipei 100, Taiwan; ^7^Graduate Institute of Medicine, College of Medicine, Kaohsiung Medical University, Kaohsiung 807, Taiwan; ^8^Department of Medical Laboratory Science and Biotechnology, China Medical University, Taichung City 404, Taiwan

## Abstract

Melanoma is an aggressive cancer with high lethality. In order to find new anticancer agents, isokotomolide A (Iso A) and secokotomolide A (Sec A) isolated from *Cinnamomum kotoense* were identified to be potential bioactive agents against human melanoma but without strong antioxidative properties. Cell proliferation assay displayed Iso A and Sec A treated in the normal human skin cells showed high viabilities. It also verified that two of them possess strong antimelanoma effect in concentration-dependent manners, especially on B16F10, A2058, MeWo, and A375 cells. Wound healing assay presented their excellent antimigratory effects. Through 3-*N*,3-*N*,6-*N*,6-*N*-Tetramethylacridine-3,6-diamine (acridine orange, AO) staining and Western blot, the autophagy induced by treatment was confirmed, including autophagy-related proteins (Atgs). By using annexin V–FITC/PI double-stain, the apoptosis was confirmed, and both components also triggered the cell cycle arrest and DNA damage. We demonstrated the correlations between the mitogen-activated protein kinase (MAPK) pathway and antimelanoma, such as caspase cascade activations. To further evaluate *in vivo* experiments, the inhibition of tumor cell growth was verified through the histopathological staining in a xenograft model. In this study, it was confirmed that Iso A and Sec A can encourage melanoma cell death via early autophagy and late apoptosis processes.

## 1. Introduction

Skin cancer is one of the most aggressive malignant tumors, which especially occurs in people with light skin. The primary reason of skin cancer is the overexposure to ultraviolet (UV) radiation [1]. Chemical mutagens, genetic susceptibility, human papillomavirus, and tobacco consumption can also lead to skin cancer. In addition, the utilization of immunosuppressive drugs such as azathioprine and cyclosporine A has been reported to result in the occurrence of skin cancer [2]. There are three skin cancers: squamous cell carcinoma (SCC), basal cell carcinoma (BCC), and malignant melanoma. BCC grows slowly and can damage the surrounding tissue, but it does not have the ability to spread to other areas. SCC is a kind of abnormal cell cancer with uncontrolled growth arising from the squamous cells. It can occur in all areas of the body and are the most common in the areas exposed to sunlight. SCC possesses the ability of metastasis and can harm other areas of the body. BCC and SCC are defined as nonmelanoma skin cancer (NMSC) [3]. Malignant melanoma is highly aggressive which develops from the pigment-containing cells known as melanocytes. Melanoma can spread rapidly through the whole body of the patient, and thus causes a very low survival rate [4].

The most effective method to prevent skin cancer is avoiding exposure to UV radiation and using sunscreen. If the skin cancer occurs, in the initial stage, it can be curable by surgical elimination, while in the advanced stage, it is usually treated by immunotherapy and chemotherapy [5]. The classical pharmacotherapy employed for the treatment of skin cancers usually includes 5-fluorouracil, imiquimod, diclofenac, and ingenol mebutate. In recent years, the phytochemicals such as quercetin, epigallocatechin-3-gallate, resveratrol, and curcumin have been applied in the melanoma treatment [6–8].

**Figure 1:**
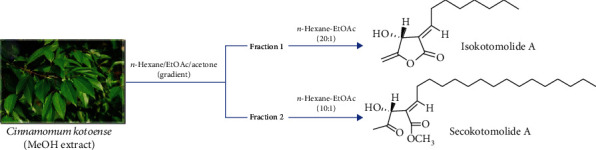
The purification and chemical structures of Iso A and Sec A.

The mitogen-activated protein kinase (MAPK) cascade activation is the center of various signaling pathways. It plays an important role in receiving the membrane receptors to convert and transmit signals into the nucleus. There are three different pathways of MAPKs, i.e., the extracellular-signal-regulated kinase (ERK), c-Jun N-terminal kinase (JNK), and p38 MAPK. The JNK pathway is known to regulate the cell growth and death, however, the mechanism of how the JNK/MAPK signaling pathway inhibits the melanoma has not been fully clarified. This study was aimed at figuring out the naturally occurring chemotherapeutic agent and evaluated their cytotoxicity on melanoma. *Cinnamomum kotoense* Kanehira is a kind of Lauraceae evergreen small tree, which is native to Lanyu Island, a small island of southeast Taiwan. The bark is used as medicine to prevent colds, pain, bleeding, and so on. It is also rich in cinnamon oil to be the main component as cinnamaldehyde and exudes fragrance to clean air. Iso A and Sec A were constitutes isolated from the leaves of *Cinnamomum kotoense* (Figure 1). A previous study had shown that Iso A was an agent capable of inducing human nonsmall lung cancer apoptosis [9]. Iso A and Sec A also exhibited cytotoxicity on Hela cells [10]. In this study, a hypothesis was proposed that both components were potential elements against the melanoma cells to investigate the possible antimelanoma mechanism. We investigated the effects of Iso A and Sec A on the suppressions in cellular proliferation, cell cycle arrest, cell migration, and the activations of autophagy and apoptosis pathways, and *in vivo* examinations.

## 2. Materials and Methods

### 2.1. Materials and Reagents

Vitamin C, ethylene diamine tetraacetic acid (EDTA), butyl hydroxy anisd (BHA), trichloroacetic acid, potassium ferricyanide, FeCl_3_, FeCl_2_·4H_2_O, 3-(4, 5-Dimethylthiazol-2-yl)-2, 5-diphenyltetrazolium bromide (MTT), dimethyl sulfoxide (DMSO), Dulbecco's modified Eagle's medium (DMEM), fetal bovine serum (FBS), Trpsin-0.5%EDTA, and phosphate-buffered saline (PBS). Antibodies against *β*-actin, cleaved caspase 3, 9, and peroxidase-conjugated anti-rabbit IgG secondary antibody were purchased from cell signaling (Beverly, MA, USA). Antibodies of B-cell lymphoma 2 (Bcl-2), JNK, Beclin-1 (Atg-6), Atg 5, 10, and 16 were purchased from ABGENT Co. (San Diego, CA, USA). Peroxidase-conjugated anti-mouse IgG secondary antibody was purchased from Pierce (Rockford, IL, USA). All the other chemicals and reagents were purchased from Sigma Chemical (St. Louis, MO, USA).

### 2.2. Extraction and Isolation of Compounds

The air-dried leaves of *Cinnamomum kotoense* (11.0 kg) were extracted with MeOH (80 L × 6) at room temperature, and the MeOH extract (201.2 g) was obtained upon the concentration under reduced pressure [9]. The MeOH extract, suspended in H_2_O (1 L), was partitioned with CHCl_3_ to give fractions soluble in CHCl_3_ and H_2_O. The CHCl_3_-soluble fraction was chromatographed over silica gel using *n*-hexane-EtOAc-acetone as an eluent to produce five fractions. Part of fraction 1 was subjected to Si gel chromatography by eluting with *n*-hexane-EtOAc, and then enriched with EtOAc to furnish 10 fractions. Fraction 1-1 was resubjected to Si gel chromatography, eluting with *n*-hexane-EtOAc, and enriched gradually with EtOAc to obtain five other fractions. Fraction 1-1-2 eluted with *n*-hexane-EtOAc was further separated using silica gel column chromatography and preparative thin-layer chromatography (TLC), giving Sec A (327 mg). Part of fraction 2 was subjected to Si gel chromatography by eluting with *n-*hexane-EtOAc and enriched with EtOAc to furnish six fractions. Fraction 2-3 was resubjected to Si gel chromatography, eluting with *n-*hexane-EtOAc, and enriching gradually with EtOAc to obtain four fractions [11]. Fraction 2-3-2 eluted with *n-*hexane-EtOAc was further separated with silica gel column chromatography and preparative TLC (*n-*hexane-EtOAc; and gave Iso A).

### 2.3. 1, 1-Diphenyl-2-Picrylhydrazyl (DPPH) Radical Scavenging Activity

DPPH is an unstable radical and has a strong absorbance at 517 nm, when DPPH is reduced by an antioxidant or combined with another free radical, its absorbance will be reduced or even disappear [11]. Thus, DPPH is applied to examine the antioxidant activity by detecting radical scavenging capability. Briefly, added 1.0 *μ*L accurate concentration (10, 50, 100, and 200 *μ*M) of Iso A or Sec A to 99 *μ*L DPPH (60 *μ*M). In addition, the positive control was vitamin C, because of its excellent antioxidant activity. We detected the absorbance value at 517 nm; the radical scavenging capability (percentage) was calculated by formula as follows:

(1) $$\begin{array}{c} \text{Radical scavenging activity}\left(\%\right)=\frac{\left(A_{\text{control}}-A_{\text{sample}}\right)}{A_{\text{control}}}\times 100\%. \end{array}$$

### 2.4. Metal Chelating Activity

Metal ion is one of the causes for the lipid peroxidation, especially ferrous ion which is prooxidant. This assay is based on the complexes of ferrous ions and ferrozine have a color reaction at 562 nm, and the lower absorbance means the better metal chelating activity [12]. 1.0 *μ*L various dosages (10, 50, 100, and 200 *μ*M) of Iso A or Sec A were added into 10 *μ*L FeCl_2_·4H_2_O (2.0 mM) to mix with 20 *μ*L ferrozine (5.0 mM), and EDTA acted as a positive control. The absorbance of the mixture was observed at 562 nm. The chelating power activity was calculated as Equation (1):

### 2.5. Reducing Power

We used the reduce power assay to test the reductive ability of Iso A and Sec A. Briefly, 85 *μ*L, 0.2 M phosphate buffer (pH 4.4), 2.5 *μ*L K_3_Fe (CN)_6_ (20%), and various suitable concentrations of samples were mixed together at 50°C for 20 min. After that, 160 *μ*L of trichloroacetic acid (TCA) (10%) was added to the mixture with the centrifugation at 3,000 rpm for 10 min to collect the supernatant (75 *μ*L). 25 uL FeCl_3_ (2%) was incubated with the supernatant for the optical absorbance measurement at 700 nm. Butyl hydroxyl anisd (BHA) was viewed as a positive control. A higher absorbance value means a better reductive activity [13].

### 2.6. Cell Cultures

Hs68, HaCaT, B16F10, MeWo, A2058, and A375 cell lines were purchased from the Bioresource Collection and Research Center (BCRC, Hsinchu, Taiwan). Six cells were kept in a cell culture dish at 37°C and 5% CO_2_ in DMEM supplemented with 10% FBS, 100 U/mL of penicillin, 100 mg/mL of streptomycin, and 0.25 *μ*g/mL of amphotericin B [10]. DMSO was dissolved stock solutions of Iso A and Sec A (both at 1.0 M), and the tested concentrations were prepared by diluting with free medium, and the final DMSO concentration was less than 1.0% to avoid from unexpected biochemical reactions.

### 2.7. Cell Proliferation Assay

The cell proliferation effects of Iso A and Sec A on normal and melanoma cells were tested by MTT assay [14]. This method was based on the property that mitochondrial succinate dehydrogenase from living cells made MTT reduced to water-insoluble blue-violet crystal formazan and deposited in the cells, and dead cells without this function. Briefly, 1 × 10^4^ cells were seeded in a 96-well microplate. After 24 h, discarded the medium, cells were treated with various concentrations (10, 25, 50, and 100 *μ*M) of Iso A and Sec A or vehicle control in a final volume of 100 *μ*L culture medium. The experimental doses were accorded to our previous tests. After cultured within 37°C, 5% CO_2_ incubator for 24 h, we replaced the medium with 100 *μ*L fresh broth contained 0.5 mg/mL MTT. The plate was kept in a 37°C incubator for 2 h. We discarded the medium to add 100 *μ*L DMSO dissolving the purple formazan crystals and gently shook the plate in darkness for 10 min. The optical absorbance values (*A*) were measured at 595 nm (BioTek, WA, USA). Cell viabilities were calculated according to the formula as follows:

(2) $$\begin{array}{c} \text{Cell viability}\left(\%\right)=\frac{\left(A_{\text{sample}}-A_{\text{blank}}\right)}{\left(A_{\text{control}}-A_{\text{blank}}\right)}\times 100\%. \end{array}$$

### 2.8. Autophagic Vacuoles Detection by Acridine Orange Staining

B16F10 cells were chosen for the further homologous study to seed in a 6-well dish at a density of1 × 10^5^, after 24 h, treated with various concentrations of Iso A and Sec A for 24 h, washed cells twice with PBS, and then incubated with serum-free medium contained 5 ug/mL acridine orange (AO, Molecular Probes, Eugene, Oregon, USA) at room temperature for 15 min [15]. We used an inverted fluorescence microscope to obtain the AO fluorescence images. AVO was stained with red color, and the nucleus DNA and cytosolic RNA were stained with green color.

### 2.9. Annexin V-FITC/PI Binding Assay to Analyze Apoptosis

To further confirm the apoptosis of Iso A and Sec A-treated malignant melanoma B16F10 cells, annexin V-fluorescein isothiocyanate (FITC)/propidium iodide (PI) double-staining was performed (Biovision, annexin V-FITC apoptosis kit) [16]. 1 × 10^5^ cells were kept in a 6-well dish and were treated with tested Iso A and Sec A concentrations (10, 25, 50, and 100 *μ*M) or vehicle control in a total volume of 100 *μ*L culture medium for 24 h. We collected the medium, washing twice with PBS to trypsinize the adherent cells. The centrifugation at 1,000 rpm at 4°C for 10 min was set to collect cells and label with annexin V–FITC/PI. The incubation was in darkness for 15 min, and the samples were analyzed by a flow cytometry (Millipore guava easyCyte HT, MA, USA).

### 2.10. DNA Damage and Cell Cycle Analysis

To identify the phase distribution of cellular DNA, cell cycle analysis was performed as described [17]. B16F10 cells were cultured at a density of 1 × 10^5^ cells in a 6-well dish, and then monolayer cells were treated with various concentrations (10, 25, 50, and 100 *μ*M) of Iso A, Sec A, or vehicle control for 24 h. Collected adherent and floating cells and washed once with precooled PBS, then fixed cells with precooled 70% ethanol overnight at 4°C. After centrifugation at 1,200 rpm for 3 min, wash the cell pellets once with precooled PBS, centrifuged again, and then stained cells with 100 *μ*g/mL RNase and 50 *μ*g/mL PI in PBS buffer, and incubated in darkness at room temperature for 30 min. The DNA contents of PI-stained cells were analyzed by flow cytometry.

### 2.11. Western Blot Analysis

1 × 10^6^ B16F10 cells were treated with Iso A, Sec A, or vehicle control for 24 h. Cells were harvested and lysed with lysis buffer (Thermo Scientific Pierce RIPA Buffer) to extract cellular proteins. The lysates were centrifuged at 1,2000 rpm for 30 min, and the protein concentrations were measured with the bicinchoninic acid (BCA) protein assay kit (Pierce, Rockford, IL, USA). Sodium dodecyl sulfate-polyacrylamide gel electrophoresis (SDS-PAGE) was performed to separate the same amounts of proteins and then electrotransferred the proteins on gel to a polyvinylidene fluoride (PVDF) membrane (PALL Life Science, Ann Arbor, MI, USA). The membrane was blocked with a blocking buffer (Pierce TOOLSpeed PLUS Blocking Reagent) and washed with TBST (Tris-buffered saline, with Tween-20, pH 8.0). The membrane was incubated with the corresponding primary antibody, shaking at 4°C overnight, washed three times with TBST and blocked again, and then incubated with secondary antibody against the corresponding primary antibody for 90 min. The signal was visualized by enhanced chemiluminescence (ECL) detection with West Femto Maximum Sensitivity Substrate kit (SuperSignal, Rockford, IL, USA) [12].

### 2.12. RNA Isolation and Extraction

The RNA was isolated and extracted by Trizol RNA isolation reagent, which could resolve cell lysates and separate RNA, DNA, and proteins. First, 1 mL Trizol reagent was increased to each and move to the 1.5 mL microtube at room temperature for 5 min. Then, 200 *μ*L BCP per mL of Trizol reagent was added and mixed intensely [18]. After incubation for 2 min, the samples were centrifuged at 14,000 × g for 20 min. The sample homogenates formed three phases, from which the aqueous phase on the top of the homogenate was move to a new Eppendorf tube. To precipitate RNA, an equal volume of isopropanol was blended. The mixture was centrifuged at 13,000 × g for 15 min, and the supernatant liquid was divided. The RNA pellet was washed with 1 mL of 75% ethanol to get rid of the residual salts. In the end, the mixture was centrifuged at 13,000 g for 5 min, and the RNA pellet was dehydrated and dissolved with 50 *μ*L diethylpyrocarbonate- (DEPC-) treated water. The concentration and quality of the RNA extracts were determined by BioTek (Lionheart Technologies, Inc.)

### 2.13. Quantitative Real-Time Polymerase Chain Reaction (qRT-PCR)

qRT-PCR is a method detected for evaluating gene expression level by measuring the cDNA products after each cycle of PCR increase. For qRT-PCR, a reactive mixture with SYBR Green Master Mix (Qiagen, Valencia, CA, USA) templates and primers was used [4]. All qRT-PCR reactions were finished by a StepOnePlus™ System. The reactions were carried out according to the following program: cDNA templates were initiated at 95°C, annealed at 65°C, and elongated at 70°C, and all steps were repeated with 40 cycles of enlargement. At the end of the annealing stage of the experiments, fluorescence acquisition was started to determine. The designed forward and reverse primers from 5′ to 3′ used in this experiment are shown in (Table S1).

### 2.14. Cell Migration Assay

The population of cellular migratory inhibition effect was examined by wound healing assay as described [15] and performed with minor modification. In brief, 5 × 10^5^ cells were seeded in 6-well plates and grown to complete confluence. We created a clear wound area on a monolayer culture with a 200 *μ*L plastic pipette tip to wash once with PBS and then treated with the samples. Afterward, we used a microscope taking photos at time intervals of 0, 6, 12, and 24 h to check the wound closure. The cell movement and migration through the wound area were calculated by the free software, ImageJ.

### 2.15. Animal Material

In this study, the application of animals complied with the guiding principles in the care and use of animals of the American physiology society was approved by the National Chung Hsing University Use Committee (IACUC: 106-111, Figure S1). BALB/c nude female mice (4-5 weeks) were purchased from BioLASCO Experimental Animal Center (Taiwan Co., Ltd) [19, 20]. The mice were housed in plexiglass cages in a temperature-controlled room (22 ± 1°C), on a 12 h/12 h light/dark schedule, and with free access to food and water (fed a standard laboratory diet). After one week, 18 mice were randomly divided into 3 groups (*n* = 6, each group): Group A, vehicle blank control; Group B, B16F10 only; and Group C, B16F10 with Iso A treatment.

### 2.16. Xenograft Tumor Assay

The performance of xenograft tumor assay was described previously with minor modifications. In brief, BALB/c nu/nu female mice were housed, and the *in vivo* experiments were performed at the animal center. Mice were implanted subcutaneously with 1 × 10^7^ of B16F10 homologous cells in 0.1 mL PBS injected subcutaneously in each mouse [19]. Mice were treated four times a week with a subcutaneous injection of Iso A (200 mg/kg) until sacrifice at day 35. The diameters of xenograft tumor were measured at 4 days intervals with Vernier calipers and calculated as (length × width^2^)/2 in mm^3^.

### 2.17. Histopathological Analyses of Xenografted Tumor

The fresh tumor tissues were made in paraffin, cut into 3 mm thick chunks, set in plastic cassettes, and immersed in 10% neutral buffered formalin for 7 days. Staining of sectioned paraffin-embedded tumor tissue with Hematoxylin and Eosin (H&E). This stain is visible for tissue processing, embedding, and sectioning. The range of Iso A-treatment shrunk the tumor weight and volume was evaluated, and the assessment of mitotic cell division in the xenografted tumor fragments was also observed via H&E images [20].

### 2.18. Statistical Analysis

Statistical data were shown as mean ± standard deviation (SD) values, and Student's test was applied to determine the difference between the control vehicle and experimental groups, ^∗^*p* < 0.05 versus control; ^∗∗^*p* < 0.01 versus control.

## 3. Results

### 3.1. Antioxidant Activity of Iso A and Sec A

Since melanomas occur on the skin and most of them are caused by UV exposure, the impacts of oxidative stresses on melanoma have attracted researchers' attentions. Antioxidants are thought to prevent UV-induced oxidative stress and DNA damage, and previous studies have shown that antioxidant intakes can prevent melanoma developments. Therefore, the antioxidant activities of Iso A and Sec A were evaluated (Table 1). At testing concentrations, two constitutes did not show good antioxidant activities, and thus it was speculated that both components did not suppress melanoma proliferation by antioxidative properties.

### 3.2. Antiproliferative Effects of Iso A and Sec A

**Figure 2:**
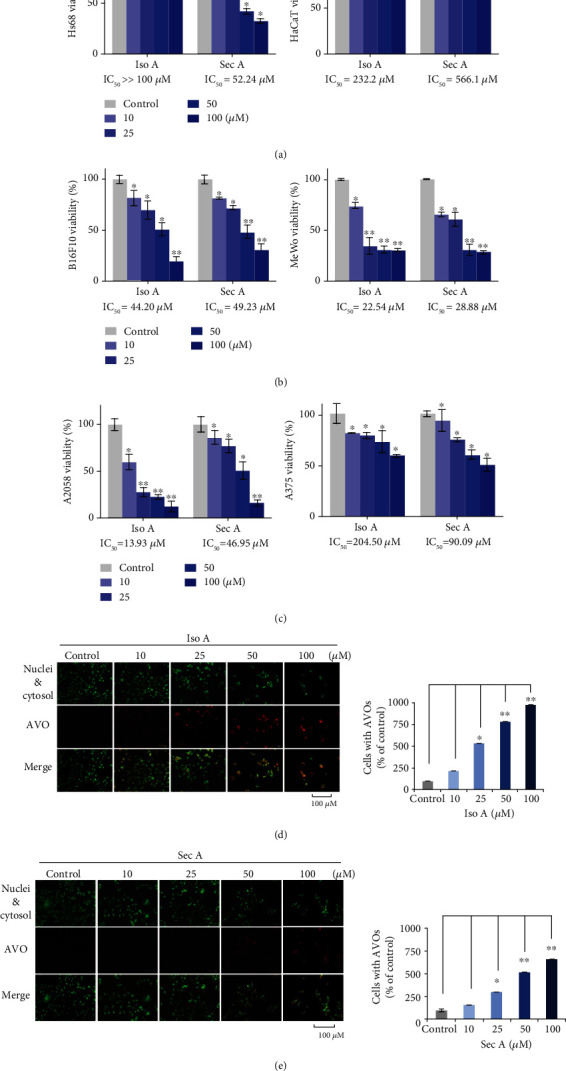
Effects of Iso A and Sec A on the normal and melanoma cells. (a) HaCaT and Hs68 proliferations after the treatments of Iso A and Sec A for 24 h. (b) Quantifications of B16F10, MeWo, A2058, and A375 cell growth inhibitions in the above conditions, respectively. (c) Fluorescent images of Iso A and Sec A-induced acidic autophagic AVOs. Cells were stained with AO to detect with a fluorescence microscopy. At least three photos were taken in various view versions, and one was chosen as an example to be demonstrated. The data represented mean values ± SD of three independent experiments performed. ^∗^*p* < 0.05 and ^∗∗^*p* < 0.01. *n* = 3.

This work was aimed at finding the potential agents with antimelanoma effects, and a good leading compound should also be safe enough for normal cells. Thus, the effectivenesses of two components was evaluated on the proliferation of normal human skin cells including fibroblast Hs68 cell line and keratinocyte HaCaT cell line.  Figure 2(a) showed the results from MTT assay of 24 h treatment at the concentrations of 0 (a vehicle control), 10, 25, 50, and 100 *μ*M, respectively. All experimental concentrations were at less than 1.0% DMSO. Iso A presented no cytotoxicity on fibroblasts and minor cytotoxicity on keratinocytes at high concentrations. Sec A demonstrated a slight cytotoxic effect on keratinocytes and fibroblast cells at concentrations of 10 and 25 *μ*M, respectively, but at the concentrations of 50 and 100 *μ*M, Sec A influenced the survival of fibroblasts. IC_50_ (the half-maximal inhibitory concentration) is the quantitative value of the potency on Iso A or Sec A in the suppression to the specific cellular viability. To evaluate the effects of two compounds on malignant melanomas, the MTT method was also applied on B16F10, MeWo, A2058, and A375 cells (Figure 2(b)). Four melanoma cells were treated with concentrations from 0 to 100 *μ*M for 24 h, respectively. The cell proliferation of melanoma cells was inhibited by both compounds in concentration-dependent manners, and the treatments illustrated excellent anticancer effects, especially on B16F10, A2058, and MeWo cells. At the concentrations of 50 and 100 *μ*M, we observed that more than half of the cells were dead. According to these findings, we chose B16F10 as our testing cell line at 25 and 100 *μ*M for the further studies.

### 3.3. The Formation of Autophagic Vacuoles (AVO) in Iso A and Sec A-Treated B16F10 Cells

To identify if Iso A and Sec A treatments will induce autophagic cell death in B16F10 cells, AO staining was performed. AO is a lysomotropic metachromatic fluorescent dye and can be used to analyze the lysosomal membrane permeability status. During the autophagy process, autophagosome fuses with lysosome to produce autolysosome, phagolysosome, and autophagolysosome. B16F10 cells were incubated with Iso A and Sec A and then labeled with AO to visualize the acidic vesicular organelles (AVOs) of acidic autophagolysosome. The cell nuclei DNA and cytosol RNA were stained with green color, and AVOs were observed in red color (Figure 2(c)). There was an obvious enhancement of red fluorescence, which indicated that testing samples can induce autophagy on the melanoma cells. In addition, the number of cells was also significantly reduced at high concentrations, pointing out the excellent cytotoxicity in dose-dependent manners.

### 3.4. Iso A and Sec A Cause Apoptotic Cell Death on B16F10 Cells

**Figure 3:**
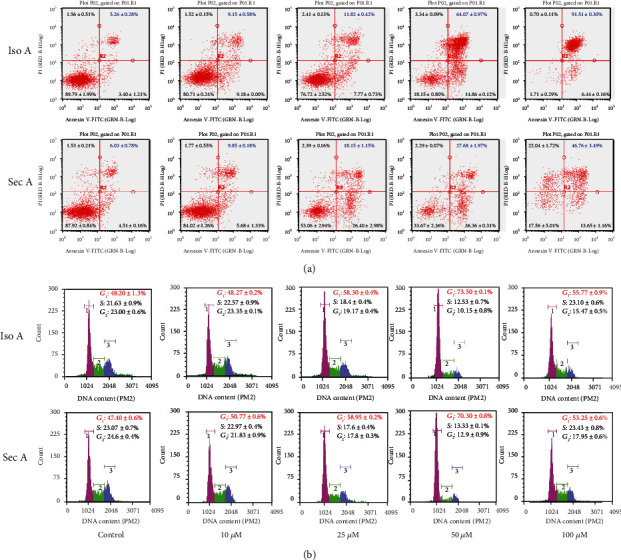
Cell apoptotic death and cycle distribution in B16F10 cells of Iso A and Sec A. (a, b) Iso A and Sec A induced cell apoptotic death. Apoptosis was analyzed by a flow cytometry after exposed to Iso A from 0 to 100 *μ*M for 24 h and stained with annexin V/PI. (c, d) Effects of Iso A and Sec A on the cell cycle distribution in B16F10 cells. The cell cycle distribution in Iso A-induced B16F10 cells was determined by the flow cytometry. Cells were administered from 0 to 100 *μ*M for 24 h. The data represented mean values ± SD of three independent experiments performed. *n* = 3.

The two-dimensional flow cytometry was employed to evaluate the cytotoxic effects of Iso A and Sec A. In the early apoptotic cells, cell membrane surface damage occurs, and the phospholipidine serine (PS) on the inner surface can be reversed to the outer membrane of the cell. Annexin V is a phospholipid-binding protein with a high affinity for phosphatidylserine and was applied to detect the early cell apoptosis. Propidium iodide (PI) is a nucleic acid dye and was used to label the late apoptotic cells and dead cells. The flow cytometry presented annexin V-FITC staining in *x*-axis and PI staining in *y*-axis. Briefly, the left lower quadrant means living cells, and the right lower and right upper quadrants mean early and late apoptotic cells, respectively. In addition, the left upper quadrant refers to necrotic cells. B16F10 cells were cultured with various concentrations of Iso A and Sec A or a vehicle control for 24 h (Figure 3(a)). The treated cells presented obvious apoptosis compared with the vehicle control. Iso A caused significant apoptotic cell death at the concentration of 50 and 100 *μ*M, and the percentage of late apoptosis cells in the group treated with 50 *μ*M Iso A was increased to 64.07 ± 0.97% and the group treated with 100 *μ*M was increased to 91.51 ± 0.30%, compared with 5.26 ± 0.28% of the control. Sec A presented a concentration-dependent manner to trigger apoptosis mechanism as Iso A to suppress melanoma growth. The group treated with 100 *μ*M Sec A induced 46.76 ± 3.49% cells to enter late apoptosis, compared with 6.03 ± 0.78% of the vehicle control group.

### 3.5. Iso A and Sec A Induce DNA Damage and Cell Cycle Arrest

The cellular DNA content and cell cycle were tested after the treatments at various concentrations of Iso A and Sec A for 24 h. B16F10 cells were harvested and washed once with PBS, fixed by precooled 70% ethanol overnight, stained by PI, and analyzed by the flow cytometry. The accumulation of the G0/G1 population is usually thought as a biomarker of DNA damage and an occurrence of apoptosis [21]. As shown in Figure 3(b), after exposure to Iso A for 24 h, the G0/G1 accumulation arose obviously, and the G2/M profile decreased. To compare with 48.20 ± 1.3% on the shape in G0/G1 phase of vehicle control, the treated groups were increased to 48.27 ± 0.2%, 58.30 ± 0.4%, 73.50 ± 0.1%, and 55.77 ± 0.9% at the concentrations of 10, 25, and 50 *μ*M, respectively. At 100 *μ*M, alive B16F10 cells were too few to be examined precisely. Sec A induced DNA damage to arise the G0/G1 accumulation which was similar to Iso A performance. We compared with the value of 47.4 ± 0.6% in G0/G1 accumulation in the control group; Sec A treatment elevated the other group values to 50.77 ± 0.6%, 58.95 ± 0.2%, and 70.30 ± 0.8% in sequence at 10, 25, and 50 *μ*M. For the same reason, at high dose, B16F10 surface structure and DNA integrity were not good to be detected. In addition, the tumor cellular viability was decreased from 10 to 100 *μ*M compared with the vehicle control group, which indirectly indicated the dose-dependent anticancer effects.

### 3.6. The Autophagy-Related mRNA and Protein Expressions

**Figure 4:**
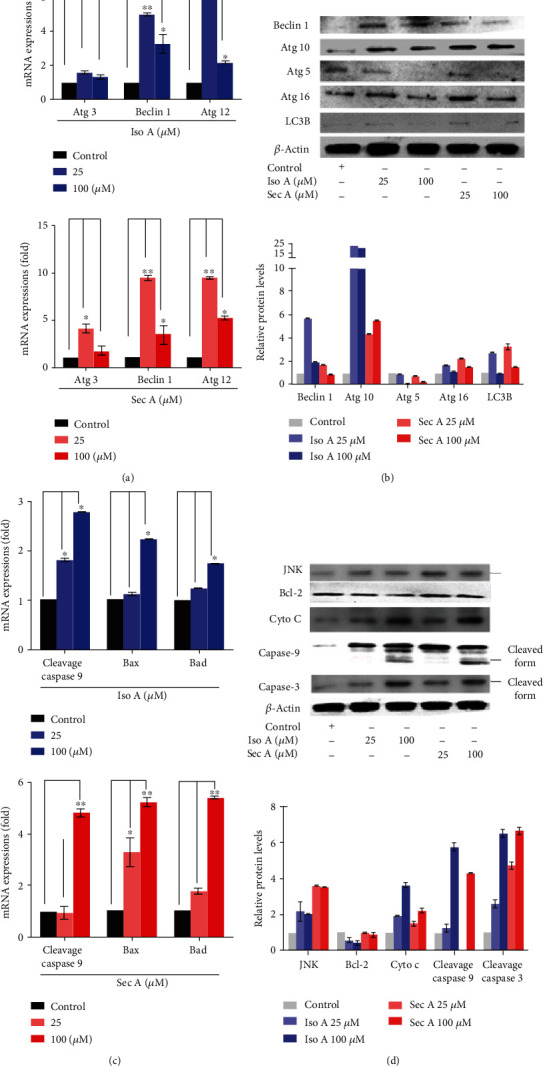
The autophagy and apoptosis-related mRNA and proteins induced by Iso A and Sec A in B16F10 cells. (a) Atg 3, 6, and 12 mRNA expression in B16F10 cells treated with different concentration (25 and 100 *μ*M) was evaluated by the quantitative polymerase chain reaction and normalized to the GAPDH gene. (b) The autophagic-related proteins Beclin 1, Atg 10, 5, and 16 expressions. *β*-Actin was viewed as an internal control. (c) The mRNA expression of caspase 9, Bax, and Bad in B16F10 cells treated with different concentrations (25 and 100 *μ*M) was evaluated by the quantitative polymerase chain reaction and normalized to the GAPDH gene. (d) The apoptosis-related proteins JNK, Bcl-2, Cytochrome c, Caspase 9, and 3 expressions in B16F10 cells with or without Iso A and Sec A treatment. *β*-Actin was viewed as an internal control. The data represented mean values ± SD of three independent experiments performed. ^∗^*p* < 0.05 and ^∗∗^*p* < 0.01. *n* = 3.

Since the AVO fluorescence enhancement after 24 h treatment was observed, quantitative real-time polymerase chain reaction (qRT-PCR) and Western blot analyses were conducted to further confirm the autophagy performance in Iso A and Sec A treated-B16F10 cells. In Figure 4(a), autophagic-related genes were examined, and we chose representative hallmarks to present this mechanism. Here, the transcriptional expressions involved in the programmed cell death signaling pathway were evaluated by the treatments of both experimental groups. It revealed that gene expressions of Atg 3, 6 (Beclin-1), and 12 were enhanced at a low dosage of 25 *μ*M (early cell death phase) and decreased at 100 *μ*M (late cell death phase). We also demonstrated typical autophagic-related proteins in Figure 4(b) after both compound treatments. Beclin-1, Atg 10, 16, and LC3B expressions were initially increased and then diminished at the concentrations of 25 and 100 *μ*M, respectively, to show a consistent phenomenon with mRNA expressions. In addition, the expression level of Atg 5 was not upregulated drastically, but diminished significantly. The results presented that at the concentration of 25 *μ*M, the expressions of mRNA and proteins were higher than that at 100 *μ*M to point out in the early phase (lose dose), melanoma triggered a programmed cell death of autophagy.

### 3.7. Examining the Apoptotic-Related mRNA and Protein Expressions

Caspases are a family of cysteinyl aspartate-requiring proteases, which can be activated by proapoptotic stimuli. To additionally investigate the molecular mechanisms underlying the induced apoptosis in the cells, qRT-PCR analysis was focused on two panels of related genes in Figure 4(c). The tested concentrations were 0 (control, with DMSO less than 1%, and PBS buffer), 25, and 100 *μ*M for Iso A and Sec A-treated groups, respectively. There was a concentration-dependent extending manner on three genes in comparison to unstimulated counterparts, including caspase 9, Bax, and Bad. Western blot analysis was performed after 24 h treatment (Figure 4(d)). Here, the transcriptional expressions involved in the programmed cell death signaling pathway were evaluated of famous apoptotic proteins, JNK, Bcl-2, cyto c, cleavage caspase 9, and 3. Both data demonstrated that at the concentrations of 25 and 100 *μ*M (in the late phase, high dosages), there were higher expressions of genes and proteins than the vehicle control group.

### 3.8. Cell Migration Inhibited by Iso A and Sec A

**Figure 5:**
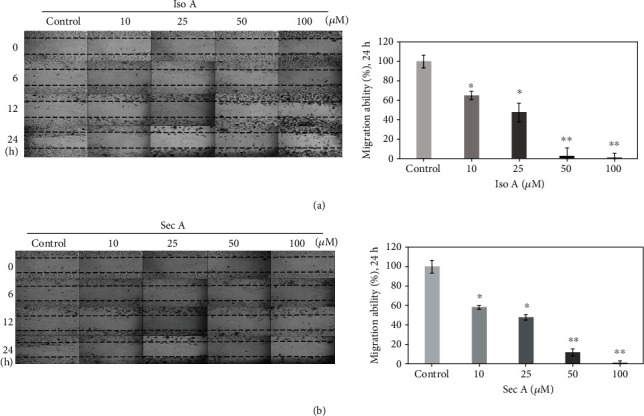
*In vitro* studies of migratory inhibition activities on B16F10 cells by Iso A and Sec A. The effects of migratory suppression were examined by wound healing assay after 24 h treatment with 0, 10, 25, 50, and 100 *μ*M, respectively. The migratory property was quantitatively measured by the software, ImageJ. The data represented mean values ± SD of three independent experiments performed. ^∗^*p* < 0.05 and ^∗∗^*p* < 0.01 (the vehicle group was compared as the control standard). *n* = 3.

The cell migration usually plays a significant role in the maintenance of normal human physiology homeostasis. However, for cancer cells, the irregular proliferation of cell migration is usually responsible for the tumor metastasis. The potential migratory inhibition of B16F10 cells by Iso A and Sec A was evaluated by cellular wound healing assay as shown in Figure 5. After the treatments of 24 h, little cells migrated into the center zone between two dash lines of two experimental groups. The cell migration capacities were weakened, and it was concluded that our compounds inhibited the melanoma cellular movements in dose-dependent manners. At the concentrations of 50 and 100 *μ*M, both compounds also had a cytotoxicity effect on B16F10 cells and influenced the survival of cells. At the concentrations of 10 and 25 *μ*M, these compounds did not show strong cytotoxicity activities on cells, but the migration abilities were greatly inhibited compared with the vehicle control. This assay indicated that Iso A and Sec A possessed migration inhibitory potentials. Besides that, we will continue to examine the suppression abilities by using transwell assay in the coming future.

### 3.9. Histopathological and Immunohistochemical Analyses

**Figure 6:**
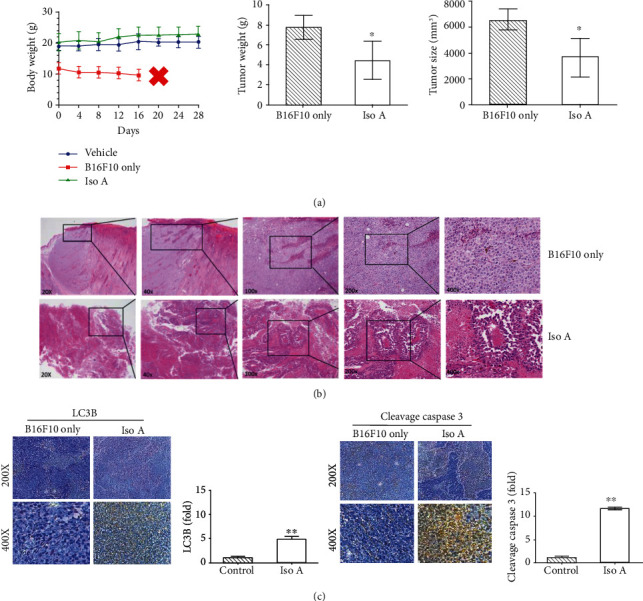
Animal test and histopathologic analysis of melanoma tumor inhibition (induced by B16F10 cells) treated with Iso A. *n* = 6. (a) Antitumor activity of Iso A in nude mice xenograft model. Mice morbidity-free survival efficacy, tumor weight, and tumor volume measurements were demonstrated after sacrifices. (b) H&E staining of tumor mass of BALB/c mice in the dorsal implant with xenograft B16F10 melanoma tumor cells of Iso A treatment. Smaller tumor mass and tumor cells expressed round to polygonal shapes with several characteristics, including melanin pigment, high mitosis, and central massive necrosis in the mouse. (c) Apoptotic protein LC3B expression and autophagic protein cleavage caspase 3 secretion in tissues treated with/without Iso A detected by immunohistochemistry. The data represented mean values ± SD of three independent experiments performed. ^∗^*p* < 0.05 and ^∗∗^*p* < 0.01.

After the above experimental studies and according to the regulations of 3R (Replacement, Reduction, and Refinement), we selected Iso A as our unique target compound to work *in vivo*. The xenograft assay showed that Iso A inhibited the melanoma B16F10 cell growth, and encouragingly, the dissected tumors were visibly smaller in the treatment groups compared to the vehicle group (Figure S2 and Figure 6(a)). We observed mice of the melanoma only group were all dead after four weeks without any therapeutic treatments; in the meantime, the other two group mice were alive with good exercise activity abilities and normal feeding properties. The data demonstrated that Iso A treatment effectively suppressed the tumor growth in the xenograft mice on tumor weight and tumor volume. Furthermore, H&E staining of the tumor tissues revealed abundant mitosis in the control group, whereas the number of mitosis-positive cells was significantly reduced in the sections from the Iso A treated mice (Figure 6(b)).

To further strengthen our research that Iso A induced autophagy and apoptosis promotes the inhibition of tumor growth in the *in vivo* examination, the immunohistochemical analyses revealed that the changes in autophagy and apoptosis were noteworthy in xenografted tumor tissues. Iso A treatment significantly increased the LC3B and cleavage caspase 3 expressions (Figure 6(c)). The quantification illustrated that the expression of cleavage caspase 3 was significantly higher than LC3B. Those results were consistent with our *in vitro* tests and indicated that Iso A induced the autophagy in the early stage and then triggered the cell death through apoptosis pathway.

## 4. Discussion

As a persuasive antimelanoma component, the agent should be harmless on normal cells and without side effects. Thus, the cytotoxicity effect on normal skin cell growth is greatly important. Therefore, the cell viability assays were performed on the normal skin cells including fibroblasts and keratinocytes [22, 23]. Iso A and Sec A are pale yellowish liquid with the molecular formulas of C_13_H_20_O_3_ and C_20_H_36_O_4_, respectively. Iso A is a butanolide and Sec A is a secobutanolide. It was found that the Sec A inhibited the cell growth of both the melanoma and normal epidermal cells and HaCat keratinocytes cells because of the long-chain chemical structure. As shown in Figure 2(a), Iso A-treated normal cells presented high cell viabilities, thus Iso A may act as a potential anticancer agent. Sec A presented little cytotoxicity on keratinocytes and mild cytotoxicity at the concentrations of 10 and 25 *μ*M on the fibroblasts, and when the concentration increased to 50 and 100 *μ*M, the cytotoxicity on the fibroblasts was also increased. Topical administration can also show good antimelanoma effect and cause less damage to normal cells.

One of the major problems with cancer treatment is the abnormal fast proliferation and metastatic of tumor cells, which also leads to an increase in recurrence and mortality. In this study, to determine the cell proliferation inhibition effects of natural products on melanoma, four melanoma cells including B16F10, A2058, A375, and MeWo were treated with Iso A and Sec A from 0 to 100 *μ*M for 24 h. As shown in Figures 2(b) and 2(c), our samples presented inhibitory activities on all four melanoma cells, and the cytotoxicity effects on B16F10, MeWo, and A2058 were relatively stronger. Previous studies have presented that the interaction between cancer cells and the extracellular matrix (ECM) plays an important role in the cell proliferation and migration [24]. Malignant melanoma is one of the most aggressive cancer, but the traditional treatment for metastatic melanoma is limited [25]. Chemotherapies such as fotemustine, dacarbazine, and temozolamide have been used for more than 35 years but are still ineffective in many ways [26]. Therefore, there is an urgent need to find new agents with antimigratory activity. In Figure 5, our results indicated that two compounds reduced the migratory ability of metastatic malignant melanoma cells. In high concentration treatment, the depression of cell migration might also contribute to the cell growth inhibition and cell death. Thus, it will be valuable to examine if Iso A and Sec A can modulate the ECM metabolism process to inhibit the metastasis and invasion of melanoma in the future work [27, 28]. In the coming future, we will continue to evaluate the migratory suppression via the transwell assay to reveal the detailed mechanism.

We further identified the roles of Atg caspase proteins in the autophagic and apoptotic cells induced by Iso A and Sec A in malignant melanoma B16F10 cells, and involved mRNA and proteins were examined. On the basis of the cell viability and cell migration assays, it was confirmed that both treatments induced the death of melanoma cells. There are three major types of programmed cell death (PCD): apoptosis, autophagy, and necrosis [29]. In this study, the first two cell death mechanisms were focused on. Autophagy is a conserved catabolic process that digests cytoplasmic components within lysosomes, and it can be triggered by organelle damage, DNA damage, and cell starvation [30]. It is thought as a survival mechanism, which accelerates the degradation of damaged cytoplasmic contents and maintains the cellular homeostasis. However, excessive active autophagy can also lead to cell death. Through AO staining (Figures 2(d) and 2(e)), the performance of autophagy induced by the experimental treatment was initially confirmed, and Western blot analysis identified the autophagic cell death. Autophagy is regulated by a set of Atg proteins. Figures 4(a) and 4(b) revealed that two components enhanced the expressions of Atg 5, 10, 6, and 16 [29].

**Figure 7:**
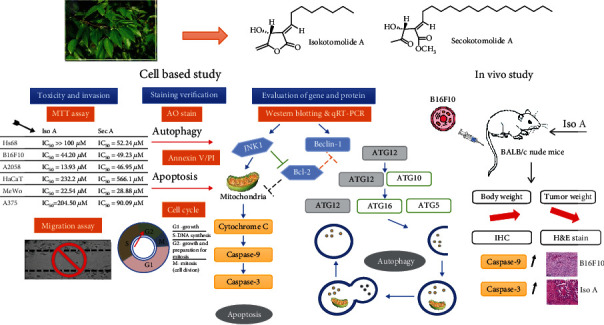
Proposed molecular mechanism diagram of *C. kotoense* components inhibit melanoma *in vitro* and *in vivo* signaling pathway.

Among three PCD types, apoptosis is considered as the principal cell death pathway [31]. A series of assays were carried out to identify that the tumor cells are induced by autophagy in the early stage, and then apoptosis is induced by the treatments after the autophagy. Annexin V-FITC/PI double staining presented that in tested concentrations, the fraction radio of late apoptosis cells which were in the right upper quadrant was significantly increased in dose-dependent manners (Figures 3(a) and 3(b)). With the purpose of blocking cell proliferation, controlling the cell cycle presents a foremost regulatory mechanism [15]. The cell cycle analysis showed that two treatments stimulated DNA damage, arrested the cell cycle, and induced G2/M accumulation (Figures 6(d) and 6(e)). Caspases play a crucial role in the process of apoptotic cascade. Apoptosis can be triggered by internal or external factors, such as cytotoxic stress, DNA damage, and growth factor withdrawal [32]. In response to those proapoptotic stimuli, the outer membrane of mitochondrial will be damaged and lose the integrity, the permeabilization of mitochondrial will be increased, and the proapoptotic protein cytochrome c will be released to accelerate the caspase activation [33]. Western blot analysis showed that both treatments triggered the release of cytochrome c, induced the activation of caspase 9, and then caspase 9 stimulated the downstream of caspase 3 (Figures 4(c) and 4(d)) [34]. JNK is an important branch of MAPK pathways, which plays a vital role in cell cycle, reproduction, apoptosis, cell stress, and other physiological and pathological processes. The results indicated that the MAPK signaling pathway might be the apoptosis reaction in those processes (Figure 7(b)). Bcl-2 is an antiapoptotic protein which can suppress the apoptosis. Previous studies have shown that Bcl-2 could inhibit autophagy by interacting with Bax/Bad and Beclin 1 [35]. The JNK-Bcl-2 pathway activation can both trigger apoptosis and autophagy [36]. Because autophagy also plays a role in protecting the cells from death stimulus, the interaction between autophagy and apoptosis in Iso A and Sec A-treated cells should undergo advanced investigation.

*In vivo* research was followed to further boost the antitumor activities of our sample by xenografted BALB/c nude mice. This study demonstrated that Iso A has lower toxicity on normal human skin cells. The treatment via subcutaneous injection in xenografted nude mice resulted in a decrease of the tumor volume during the time course [37, 38]. Histopathological H&E staining of the tumor tissues demonstrated that there was abundant mitosis in the control group; however, the dorsal implant massive necrosis phenomenon was identified in the tumor sections treated with Iso A (Figures 6(b) and 6(c)). Smaller tumor mass and tumor cells expressed round to polygonal shapes with melanin pigment, advanced mitosis, and central massive necrosis within the xenografted mice. In summary, *in vitro* and *in vivo* examinations confirmed that Iso A and Sec A treatments inhibit the melanoma growth and metastasis effectively in Figure 7.

## 5. Conclusions

Based on the above results, we targeted Iso A and Sec A isolated from *Cinnamomum kotoense* from a variety of compounds screening with the purpose of melanoma therapy. Both Iso A and Sec A showed biofunctions that induced autophagy in the early stage and induced the cell death through apoptosis in human malignant melanoma and showed a little cytotoxicity on normal skin cells. Histopathological staining and immunohistochemical staining demonstrated that the tumor cell inhibited by Iso A. To our best knowledge, this is the first study of Iso A and Sec A to be potential natural herbal components for human melanoma chemotherapy. Abbreviation within figures: Iso A: isokotomolide A; Sec A: secokotomolide A.

## Figures and Tables

**Table 1:** The antioxidant activity assays of Iso A and Sec A.

	DPPH radical scavenging ability (%)				Metal chelating activity (%)				Reducing power (OD_700_)			
Compounds (*μ*M)	10	50	100	200	10	50	100	200	10	50	100	200
Iso A	≦5.0	≦5.0	≦10.0	≦15.0	≦5.0	≦5.0	≦10.0	≦15.0	0.099 ± 0.003	0.099 ± 0.004	0.103 ± 0.002	0.127 ± 0.003
Sec A	≦5.0	≦5.0	≦10.0	19.98 ± 1.62	≦5.0	≦5.0	≦10.0	≦15.0	0.095 ± 0.003	0.101 ± 0.005	0.104 ± 0.003	0.114 ± 0.001
Vitamin C*^a^*	—	—	84.95 ± 1.86	—	—	—	—	—	—	—	—	—
EDTA*^b^*	—	—	—	—	—	—	92.66 ± 6.49	—	—	—	—	—
BHA *^c^*	—	—	—	—	—	—	—	—	—	—	0.736 ± 0.008	—

^a^Vitamin C was the positive control of DPPH radical scavenging capacity assay with concentration at 100 *μ*M; ^b^EDTA was the positive control of metal chelating activity assay with concentration of at *μ*M; ^c^BHA was the positive control of reducing power assay with concentration at 100 *μ*M.

## Data Availability

The datasets generated during and/or analysed during the current study are available from the corresponding author on reasonable request.

## Supplementary Materials

Supplementary Materials Table S1: primers were used for qRT-PCR to analyze the apoptotic and antiapoptotic gene expressions. Figure S1: IACUC certificate of animal examinations (IACUC number: 106-111). Figure S2: antitumor activity of Iso A in the xenograft nude mice model via B16F10 melanoma cell model.
