# Supplementary material for: Isokotomolide A from Cinnamomum kotoense Induce Melanoma Autophagy and Apoptosis In Vivo and In Vitro
Source: Oxid Med Cell Longev. 2020 Sep 26;2020:3425147. doi: 10.1155/2020/3425147 (PMC7537700; doi:10.1155/2020/3425147)
Supplement: Supplementary Materials — Table S1: primers were used for qRT-PCR to analyze the apoptotic and antiapoptotic gene expressions. Figure S1: IACUC certificate of animal examinations (IACUC number: 106-111). Figure S2: antitumor activity of Iso A in the xenograft nude mice model via B16F10 melanoma cell model. [file 3425147.f1.doc]

Supplementary Material

**Isokotomolide A from *Cinnamomum kotoense* induce melanoma autophagy and apoptosis *in vivo* and *in vitro***

Jian Li ^1, †^, Chung-Yi Chen ^2, †^, Jyun Yin Huang ^3^, Lin Wang ^4^, Zixuan Xu ^1^, Wenyi Kang ^5^, Miao-Hsia Lin^6^ and Hui-Min David Wang ^1,3,7,8^, *

^1^ College of Food and Biological Engineering, Jimei University, Xiamen, 361021, PR China; [lijian2013@jmu.edu.cn](mailto:lijian2013@jmu.edu.cn) ; xuzixuan9509@163.com

^2^ Department of Nutrition and Health Science, School of Medical and Health Sciences, Fooyin University, Kaohsiung 831, Taiwan; [XX377@fy.edu.tw](mailto:XX377@fy.edu.tw)

^3^ Graduate Institute of Biomedical Engineering, National Chung Hsing University, Taichung 402, Taiwan; [dsst50802felix@gmail.com](mailto:dsst50802felix@gmail.com)

^4^ College of Chemistry & Pharmacy, Northwest A&F University, Yangling, Shaanxi 712100, PR China; [wanglin0317@nwsuaf.edu.cn](mailto:wanglin0317@nwsuaf.edu.cn)

^5^ Joint International Research Laboratory of Food & Medicine Resource Function, Henan Province, Henan University, Kaifeng 475004, PR China; [kangweny@hotmail.com](mailto:kangweny@hotmail.com)

^6^ Graduate Institute of Microbiology, College of Medicine National Taiwan University, Taipei 100, Taiwan; miaohsialin1012@ntu.edu.tw

^7^ Graduate Institute of Medicine, College of Medicine, Kaohsiung Medical University, Kaohsiung 807, Taiwan; [davidw@dagon.nchu.edu.tw](mailto:davidw@dagon.nchu.edu.tw)

^8^ Department of Medical Laboratory Science and Biotechnology, China Medical University, Taichung City 404, Taiwan; [davidw@dagon.nchu.edu.tw](mailto:davidw@dagon.nchu.edu.tw)

† These authors contributed equally to this work.

***** Correspondence: [davidw@dagon.nchu.edu.tw](mailto:davidw@dagon.nchu.edu.tw); Tel.: 886-4-22840733#651

**Table S1.** Primers were used for qRT-PCR to analyze the apoptotic and anti-apoptotic gene expressions.

| Bax  Forward: 5′- GAG AGG TCT TTT TCC GAG TGG-3′  Reverse: 5′- GGA GGA AGT CCA ATGTCC AG -3′  Bad  Forward: 5′-GCGGGAATAAGTACCAGACCAT-3′  Reverse: 5′-TGCAGGCGAGACAGATTTG-3′  Caspase-9  Forward: 5′-AGGCACCGGAGAGAGAAAG-3′  Reverse: 5′-GTTTCCTGTTGCTGTGAGCTT-3′  ATG-3  Forward: 5′-CCGGTCCTCAAGGAATCAAA-3′  Reverse: 5′-GGACAGTGGTGGACTAAGTGATCTC -3′  ATG-12  Forward: 5′-CCAAGGACTCATTGACTTCATCAA-3′  Reverse: 5′-GCCAAAACACTCATATAGAGTTCCAA -3′  ATG-6  Forward: 5′-TCTGGACTTGTGTGCAGCAGTT -3′  Reverse: 5′-TTGCCTTTCTCCACGTCCAT -3′ |
| --- |

**Figure S1.** IACUC certificate of animal examinations (IACUC number: 106-111).


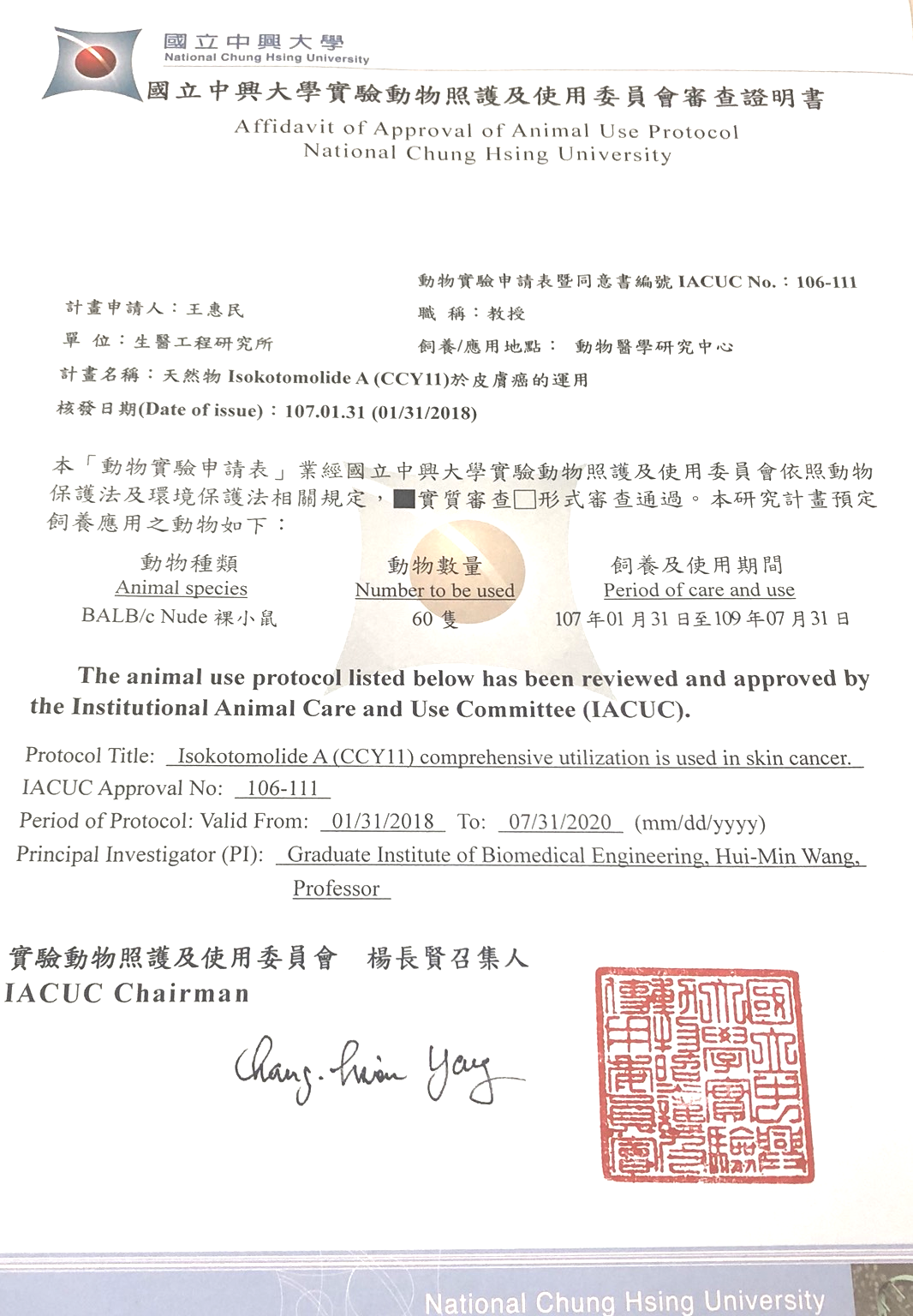


**Figure S2.** Antitumor activity of Iso A in the xenograft nude mice model via B16F10 melanoma cell model.
